# Who Reads an American Book?

**Published:** 1874-12

**Authors:** 


WHO READS AN AMERICAN BOOK?—It seems evident that a good many people read *some* American books, as we learn from a member of the American Publishing Company of Hartford that the people of this country had paid one million dollars for Mark Twain's various books before the first one—"The Innocents Abroad"—had been published five years. The same cannot be said of the works of any other author, and is an indication of the high esteem in which the writings of this inimitable humorist are held.

